# Elevated Serum Levels of Homocysteine as an Early Prognostic Factor of Psychiatric Disorders in Children and Adolescents

**DOI:** 10.1155/2012/373261

**Published:** 2012-10-02

**Authors:** Laura Kevere, Santa Purvina, Daiga Bauze, Marcis Zeibarts, Raisa Andrezina, Arnis Rizevs, Sergejs Jelisejevs, Linda Piekuse, Madara Kreile, Indulis Purvins

**Affiliations:** ^1^Department of Psychiatry and Narcology, Riga Stradins University, 215-27 Maskavas Street, 1019 Riga, Latvia; ^2^Department of Internal Diseases, Riga Stradins University (RSU), 13 Pilsonu Street, 1002 Riga, Latvia; ^3^Children's Psychiatric Hospital BKUS “Gailezers”, 20 Juglas Street, 1079 Riga, Latvia; ^4^Scientific Laboratory of Molecular Genetics, Riga Stradins University, 16 Dzirciema Street, 1007 Riga, Latvia

## Abstract

*Background and Goal*. The aim was to examine the serum levels of homocysteine (Hcy) and their associations with the methylenetetrahydrofolate reductase (*MTHFR*) gene C677T polymorphism in patients with schizophrenia and mood disorders as well as controls. *Materials and Methods*. There were 198 patients: 82 with schizophrenia spectrum disorders, 22 with mood disorders, and 94 controls. The level of Hcy was determined by an isocratic high-performance liquid chromatography system. *MTHFR* C677T polymorphism was analysed using the restriction fragment length polymorphism-polymerase chain reaction method. *Results*. The average level of Hcy was 11.94 ± 5.6 **μ**mol/L for patients with schizophrenia, 11.65 ± 3.3 **μ**mol/L for patients with affective disorders, versus 6.80 ± 2.93 **μ**mol/L in a control. The highest level of Hcy has been observed in patients with episodic-recurrent course of schizophrenia (11.30 ± 7.74 **μ**mol/L), paranoid schizophrenia continuous (12.76 ± 5.25 **μ**mol/L), and in patients with affective disorders (11.65 ± 3.26 **μ**mol/L). An association between the *MTHFR* gene C677T polymorphism and Hcy level was found by linear regression analysis (*r* = 1.41, *P* = 0.029). *Conclusions*. The data indicate a link between Hcy levels and schizophrenia and mood disorders. No associations between the level of Hcy in patients with schizophrenia and mood disorders and the *MTHFR* C677T polymorphism were found.

## 1. Introduction

Hcy was first described by Butz and du Vigneud in 1932 [1]. They obtained the product by treating methionine with a concentrated acid.

Three enzymes are directly involved in the Hcy metabolism: methionine synthase (MS), betaine homocysteine methyltransferase, and cystathionine *β*-synthase. Several other enzymes are indirectly involved. Vitamins B_6_ and B_12_ are cofactors to these enzymes, and folate is a substrate in the MS-mediated reaction [2]. Deficiencies in vitamins B_6_, B_12_, and folate can lead to high Hcy levels. Supplementation with pyridoxine, folic acid, B_12_, or folic acid, respectively, reduces the concentration of Hcy in the bloodstream.

Methionine is the immediate precursor of S-adenosylmethionine, the methyl donor of numerous methylation reactions in the brain, including many that are directly involved in the synthesis and metabolism of monoamines such as dopamine, norepinephrine, and serotonin [3]. This suggests that an association between elevated Hcy and schizophrenia is biologically plausible.

Another way of investigating the association between Hcy and mental disorders is via the *MTHFR* gene. *MTHFR* converts 5,10-methylenetetrahydrofolate to 5-methyltetrahydrofolate, which is needed for the remethylation of Hcy to methionine [4].

MTHFR is a critical component of the 1-carbon cycle, and *MTHFR *gene C677T polymorphism affects nucleotide synthesis and DNA methylation. This gives a plausible biological explanation for potential associations between genetic variation in folate metabolism and both depression and schizophrenia [5].

Previous findings indicate that the risk of suffering from psychiatric illness is correlated with elevated levels of Hcy [6]. Men with *MTHFR* gene C677T polymorphism are at greater risk of schizophrenia than women; women are at greater risk of bipolar affective disorders (BADs) [7].

Elevated Hcy is associated with an increased risk of occlusive vascular disease, birth defects (neural tube defects), complications during pregnancy, and psychiatric disorders (major unipolar depression, anxiety disorders, BADs, and in a gender-specific manner, schizophrenia) [8].

A high level of prenatal Hcy is a valid risk factor for schizophrenia and affective disorders, because Hcy is a partial antagonist of N-methyl-D-aspartate (NMDA) receptors. This antagonism can lead to the dysfunction of NMDA receptors in these patients. It is associated with changes in glycine concentration, which result in disturbances of the functioning of the placenta and pregnancy complications [2].

Hcy influences the foetus in the early stages, through disordered brain structure development and function, and/or through placenta vascularization disorders, which reduces the oxygen feed to the foetus [2].

Hcy and Hcy-acid have the ability to increase intracellular calcium levels of ions and active oxygen compounds within the cerebellum of rats as NMDA. NMDA rouses premature apoptosis of cells. These mechanisms indicate the neurotoxic impact of Hcy and its derivatives [9].

Previous studies indicate that elevated Hcy presents conditions of stress in wefts, followed by an increase in the permeability of the haematoencephalic barrier for neurotoxic materials [10, 11]. Other reports show that young men with BADs have a much higher level of Hcy than healthy people. Hcy is highest in those with more severe BAD diagnoses. Hcy may be elevated for women in all age groups but is not as high as it is for men [12–14]. Based on these results, another study found a possible association between BAD and schizophrenia with the 1p36.3 *MTHFR* locus [15]. Deterioration of clinical symptoms is observed as a result, and neurodegenerative occurrences tend to become chronic.

Based on these findings, it is important to establish which mental disorders are linked with changes of Hcy levels in blood plasma, and whether the changes in Hcy concentration depend on a clinical state of mental disorder (e.g., whether the disorder is in the early, middle, or late stage of progression, or in remission).

The aim of our study was to examine the serum levels of Hcy and determine whether they may be linked to *MTHFR *gene polymorphism and increased frequency of the TT genotype in patients with schizophrenia and affective spectrum disorders, compared with controls. We also sought to determine the clinical specificity of these disorders.

## 2. Materials and Methods

The investigation was carried out in the Department of Pharmacology at Riga Stradiņš University (RSU), Latvia, and in the Riga's Children's Psychiatric Hospital. All patients signed an informed consent form (for children, consent was given by one parent), issued accordance with RSU Medical Ethics Committee requirements.

One hundred and four patients from the Children's Psychiatric Hospital participated in the study. Patients were divided into four diagnostic groups, based on ICD-10 diagnostic criteria (ICD-10 Classification of Mental and Behavioural Disorders: Diagnostic Criteria for Research) [16] and their current health. Each diagnosis and clinical status was coded according to its severity and the course of the disease. The groups were classified as follows: group 1 (*n* = 18), paranoid schizophrenia (continuous); group 2 (*n* = 37), paranoid schizophrenia (episodic with residual symptoms); group 3 (*n* = 27), simple schizophrenia; group 4 (*n* = 22), affective spectrum disorders (depressive episode, recurrent depressive disorders with or without associated anxiety); group 5 (*n* = 94), control group of children and adolescents (healthy) (Table 1).

Healthy children, aged 3–17, were selected from kindergartens and high schools for the control group. A wide age range was used because while the parents of most of the younger children consented to blood biochemical and genetic analyses being performed, the majority of the parents of the teenagers and the teenagers themselves did not agree to such analyses. This refusal appears to be related to the fear and stigma of a prognostic genetic or mental illness being diagnosed.

Demographic characteristics of the patient groups are described in Table 2. Patients with severe somatic pathology (e.g., renal insufficiency, usage of glucocorticoids), evidence of genetic disease, and those who did not consent to participate, or whose parents or guardians refused consent, were not included in the study.

The patients' treatment regimen, designated by their attending physician, was not changed. Medications were administered in accordance with the set indications and dosing principles, including the evaluation of both contra-indications and possible side effects.

The data were registered in a special form and included information about family anamnesis, childhood, the onset of the disease, its process and individuality, all previous therapies and their results, and the current treatment and its effectiveness.

The levels of vitamin B_12_ and folic acid in the blood were analysed in the Laboratory of *Nacionalais Medicinas Serviss* (NMS). The level of Hcy in the blood was analysed in the Research Laboratory of Pharmacology Department of RSU with an isocratic high-performance liquid chromatography (HPLC) system with fluorometric detection (*Shimadzu LC-20*,* model RF-10AxL*). Sample preparation and chromatographic separation were performed in accordance with the recommendations of the commercially available *Chromsystems GmbH *(Germany) kit for HPLC analyses of Hcy in plasma.

The reduction step for releasing Hcy from its protein-bound state, protein precipitation, and precolumn derivatization followed by HPLC separation and fluorescent detection is the most widely applied technique [4].

During the research, none of the patients took vitamins—neither dietary supplements nor vitamin preparations. All samples were taken early in the morning on an empty stomach.

Genetic studies involving DNA isolations were performed in the RSU Scientific Laboratory of Molecular Genetics. The standard phenol-chloroform method was used to isolate DNA from venous blood [20]. *MTHFR* C677T (rs1801133) polymorphism was analysed by the restriction fragment length polymorphism-polymerase chain reaction (RFLP-PCR) analyses adapted method [17]. PCR was performed in a total volume of 20 *μ*L, containing 2 *μ*L 10× Taq buffer with (NH_4_)_2_SO_4_ (Fermentas), 1.6 *μ*L 25 mM MgCl_2_, 0.5 *μ*L 10 mM dNTP, 2.5 U recombinant Taq polymerase (Fermentas), and 5 *μ*L of the following 10 pmol/*μ*L primers: *MTHFR*-PF 5′TGAAGGAGAAAGGTGTCTGCGGGA3′ and *MTHFR*-P5′ AGGACGGTGCGGTGAGAGTG3′. The reaction was carried out in a TProfessional Thermocycler (Biometra). Following denaturation at 95°C for 3 min, 30 cycles at 94°C, 65°C, and 72°C for 1 min each were performed. A final extension step at 72°C for 10 min concluded the reaction. The 198-bp PCR product (4 *μ*L) was digested with restriction enzyme *Hin*fI (Fermentas) at 37°C for 18 h. *Hin*fI can recognize the cytosine to thymine substitution and formed 176 bp and 22 bp long fragments. The *Hin*fI-treated PCR fragments were analysed by 6% polyacrylamide gel electrophoresis at 180 V for 40 min, stained with ethidium bromide, and visualized under UV light. Direct sequencing (ABI Prism 300 Genetic analyser, using Big Dye v.3.1. kit (Applied Biosystems)) was used for some of the DNA samples. In all cases, genotypes corresponded to those after RFLP analysis.

## 3. Statistical Analysis

Several methods and statistical indicators were used for data analysis: mean value, average standard deviation, and average standard errors.

The validity of the difference between the average measurements of the two groups was estimated with the Student's *t*-test, with *P* < 0.05 set as the significance level.

The data were analysed using the following Stata data analysis tools: regression statistics, two-sample *t*-test with equal variances, Bartlett's test for equal variances, the and Bonferroni test. Pearson correlation coefficient *r*, also called linear or product-moment correlation, was used to determine the extent to which values of two variables were “proportional” to each other.

## 4. Results

Vitamin B_12_ and folic acid levels were found to be within the normal ranges for all patients. The correlation of Hcy concentration between the control group and psychiatric disorders is described in Table 3.

**Figure 1:**
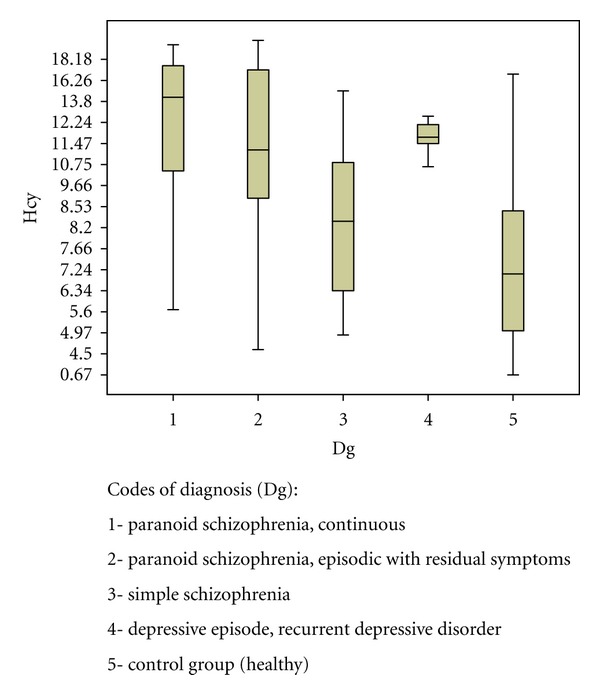
The mean level of Hcy (*μ*mol/L) in patients with affective disorders and schizophrenia spectrum disorders.

It has been found out that the highest level of Hcy in the group of schizophrenia spectrum disorders was observed in patients with paranoid schizophrenia-continuous (12.76±5.25 *μ*mol/L) and episodic-recurrent process of disease (11.30±7.75 *μ*mol/L) (*r* = − 0.56; *P* < 0.01). For the group with the affective disorders, the highest level was observed in patients with depressive symptoms of anxiety and patients with mixed affective disorders (*r* = −0.58; *P* < 0.01). The lowest level of Hcy was observed in patients with simple schizophrenia and depression without anxiety (8.47 ± 3.26 *μ*mol/L and 9.25 *μ*mol/L, resp.) (Figure 1). 

In the genetic analysis, patients were divided into three groups, depending on the existing genotype for the T allele: CC (healthy), CT (heterozygous), and TT (homozygous). Genetic analysis was completed for 180 patients: 80 patients had the CC genotype, 92 patients had the CT genotype, and only eight had the TT genotype. No genotype prevalence was observed in either of the studied patient groups (*P* < 0.01). An association between the *MTHFR* gene C677T polymorphism and Hcy level was found by linear regression analysis (*r* = 1.41, *P* = 0.029).

## 5. Discussion

Research studies have not yet convincingly demonstrated the role of Hcy in the pathogenesis of mental disorders. The findings reflect partial, episodic, and sometimes even weak clinical correlations between hyperhomocysteinemia and psychiatric disease. Replications of studies do not always support this correlation.

If schizophrenia begins before the age of 12, it is called juvenile schizophrenia. It occurs in one out of 10,000 children, but the incidence of schizophrenia and schizophrenia-related disorders rises to 1-2 out of 1000 in adolescents [21]. The fact that schizophrenia may present before puberty suggests that the cause of the disease may be a neurodevelopmental abnormality [18].

When the onset of schizophrenia occurs in childhood, it is commonly observed that the child's relatives are also likely to have a form of schizophrenia, even though they may not be diagnosed until adulthood [19].

Premature onset is more common for men and predominates between the ages of 11 and 14. Women most frequently become ill after puberty. Therefore, men more frequently have the continuous form of schizophrenia, while women are more likely to have an episodic remitting form, with affective disorders in the acute stage of the disorder. This is consistent with the fact that the process of schizophrenia is different in different age groups: younger children typically have continuous slowly progressive schizophrenia, while older children (prepubescent and pubescent) tend to have an episodic remitting course [22, 23]. More boys than girls have schizophrenia, particularly at younger ages [22, 23]. In our study, it was observed that the boys had the illness earlier and that very often the progress of the illness was continuous, whereas the girls become ill later and the progress of the illness was more often characterized by episodic intensifications and remissions. When schizophrenia occurs in childhood or adolescence, the manifestations of prodrome—development disorders or social functioning disorders—can almost always be observed. Disorders of the nervous system generate speech and language development disorders, as well as awkwardness, inattentiveness, and demoted IQ level (usually around 90) [24]. More specific cognitive deficiencies, including a decline in working memory and disorders of attention concentration, are observed in early schizophrenia [24].

Affective disorders are common in those clinically diagnosed with schizoaffective disorders, episodic schizophrenia, and continued paranoid schizophrenia.

It has been established that increased Hcy levels are commonly linked with the *MTHFR* gene TT genotype. This results in brain development disorders; the brain becomes increasingly sensitive to exotoxin, which creates a predisposition for later schizophrenia development [25]. This theory is supported by the fact that elevated Hcy levels during pregnancy are considered to be a risk factor for nervous tube development anomalies.

The *MTHFR* gene is located at the end of the short arm of chromosome 1 (1p36.3). Methylenetetrahydrofolate reductase plays a central role in folate metabolism by irreversibly converting 5,10-methylenetetrahydrofolate to 5-methylenetetrahydrofolate, the predominant circulating form of folate. 5-methylenetetrahydrofolate donates a methyl group to Hcy in the generation of S-adenosylmethionine, a major source of methyl groups in the brain [25].

Two common single *MTHFR* nucleotide polymorphisms have been reported: a C→T transition at nucleotide 766 in exon 4 (rs1801133) and an A→C transversion in exon 7 at position 1298 (rs1801131). Both of these polymorphisms are functional and result in diminished enzyme activity. For the C677T polymorphism, homozygote variants have 30% more enzyme activity than homozygotes for the wild-type C allele, while heterozygotes retain 65% of wild-type MTHFR enzyme activity [26]. The consequences of the C677T polymorphism have been demonstrated in population studies, where lower levels of red blood cell folate, plasma folate, and vitamin B_12_ have been reported among healthy persons with genotype 677 TT as well as among persons with other genotypes [26].

There was an increased risk of schizophrenia among homozygote variants (TT) in one study, although the reliability of the statistical data was low [27]. Subjects with schizophrenia showed a significantly increased frequency of the T allele. A cumulative meta-analysis showed that a moderate and significant association between schizophrenia and *MTHFR* C677T remained over time. However, no association between *MTHFR *C677T and anxiety disorders was found [28].

To date, no studies have linked hyperhomocysteinemia with psychiatric disorders in children and adolescents. Some studies have explored the associations between Hcy level and schizophrenia in adolescents by using numerically small groups of patients (aged 14–21 years). It is clear that Hcy level is higher in patients with schizophrenia than in healthy control-group patients; however, this concurrence has been observed only in boys [28].

In our study, significantly higher levels of Hcy were observed during the onset and exacerbation of the disease. This leads us to suggest that there is no relationship between Hcy and schizophrenia spectrum disorders per se but that a relationship exists between Hcy and the current affective state, in respect to the basic diagnosis.

In our study, no relationship was found between *MTHFR* gene homozygous (TT) or heterozygous (CT) genotype variants and any form of schizophrenia. Similarly, no role of the TT genotype in the elevated level of Hcy was observed.

## 6. Conclusions

Our data clearly indicate a potential link between the levels of Hcy and psychiatric disorders (schizophrenia and mood disorders). It can be concluded that the increased level of Hcy in schizophrenia and mood disorders is associated with affect and the course of the disease. Therefore, the results support the hypothesis that Hcy may be one of the risk factors of schizophrenia and mood disorders.

The data do not suggest a more frequent occurrence of any *MTHFR* gene C677T genotype (CC, CT, or TT) for any form of schizophrenia or mood disorder. Nevertheless, the collection of more precise genetic data, from a larger number of patients, is desirable.

## Figures and Tables

**Table 1:** Clinical characteristic of patients groups [17–19].

	Development	Clinical characteristic	Affective disorders	Cognitive disorders	Therapy	Prognosis
Continuous course Sch	Gradual	Relatively stable, often paranoid delusions, usually accompanied by hallucinations, particularly of the auditory variety, and perceptual disturbances. Volition and speech, and catatonic symptoms are either absent or relatively inconspicuous	Disturbances of affect. Affects that are inappropriate or blunted	Clear consciousness and intellectual capacity are usually maintained although certain cognitive deficits may evolve in the course of time	Often lasting usage of several antipsychotic medicine	Bad, progressing course with defect becoming deeper, resistance of therapy, disability
Episodic course Sch	Acute	Episodic with progressive or stable deficit, in the periods of exacerbation of disease symptoms like a continuous course Sch	Anxiety, fears	Early onset—have more significant deficits in measures of IQ, memory, and tests of perceptuomotor skills. Disturbances of working memory and attention	Aggravation therapy—commonly 2 antipsychotic medicaments, remissions—monotherapy	Bad, progressing course with defect becoming deeper, resistance of therapy, disability
Simple schizophrenia	Gradual	Simple schizophrenia is characterised with an insidious but progressive development of oddities of conduct, inability to meet the demands of society, and decline in total performance. The characteristic negative features of residual schizophrenia develop without being preceded by any overt psychotic symptoms	Cold or inappropriate affect, blunting of affect, and loss of volition	Early onset—have more significant deficits in measures of IQ, memory, and tests of perceptuomotor skills. Disturbances of working memory and attention	DRAs monotherapy, often combinations with AEDs	Bad. Gradual inellectual skills ↓, emotional disorders, formation of identity defect
Affective disorders—depressive episode, recurrent depressive disorders (with or without associated anxiety)	Subacute/ acute	Depression—the patient suffers from lowering of mood, reduction of energy, and decrease in activity. Capacity for enjoyment, interest, and marked tiredness after even minimum effort is common. Sleep is usually disturbed and appetite diminished. Self-esteem and self-confidence are almost always reduced, and, even in the mild form, some ideas of guilt or worthlessness are often present. The lowered mood varies little from day to day, is unresponsive to circumstances, and may be accompanied by the so-called “somatic” symptoms, such as loss of interest and pleasurable feelings, waking in the morning several hours before the usual time, depression worst in the morning, marked psychomotor retardation, agitation, loss of appetite, weight loss, and loss of libido		Hard to concentrate, slow thinking rate	Antidepressant ± AEDs	Good, in case of duly treatment
					

Sch: schizophrenia; IQ: intelligence quotient; DRAs: dopamine receptor antagonists; AEDs: antiepileptic drugs.

**Table 2:** Demographic characteristics of patients groups.

	Schizophrenia	Affective disorders	Control group
Gender			
Male	50 (61%)	6 (27%)	54 (57%)
Female	32 (39%)	16 (33%)	40 (43%)
Age (years)			
3–7	0	0	34 (36%)
7–12	12 (15%)	5 (23%)	26 (28%)
12–15	23 (28%)	6 (27%)	12 (13%)
15–18	47 (57%)	11 (50%)	22 (23%)
Duration of illness (years)			
1	37 (45%)	11 (50%)	0
2-3	24 (29%)	8 (36%)	0
>3	21 (26%)	3 (14%)	0
No illness	0	0	94 (100%)

**Table 3:** Correlation (*r*) of Hcy concentration between control group and psychiatric disorder groups.

Diagnosis	Control group	
	Pearson's correlation	*t* test
Schizophrenia spectrum disorders	−.46	<0.01
Paranoid schizophrenia, continuous	−.58	<0.01
Paranoid schizophrenia, episodic with residual symptoms	−.56	<0.01
Simple schizophrenia	−.19	<0.01
Patients with mood disorders	−.45	<0.01

The *t*-test is used to evaluate the differences in means between two groups.

Pearson correlation coefficient *r*, also called linear or product-moment correlation, is used to determine the extent to which values of two variables are “proportional” to each other.
